# Preparation and Properties of Cyanobacteria-Based Carbon Quantum Dots/Polyvinyl Alcohol/ Nanocellulose Composite

**DOI:** 10.3390/polym12051143

**Published:** 2020-05-17

**Authors:** Li Xu, Ying Li, Shiyu Gao, Yue Niu, Huaxuan Liu, Changtong Mei, Jiabin Cai, Changyan Xu

**Affiliations:** 1 College of Materials Science and Engineering, Nanjing Forestry University, Nanjing 210037, China; ly1728705223@icloud.com (Y.L.); gaoshiyu@njfu.edu.cn (S.G.); niuyue5186@163.com (Y.N.); alucinda@163.com (H.L.); mei@njfu.edu.cn (C.M.); nldfloor@163.com (J.C.); 2 Jiangsu Co-Innovation Center of Efficient Processing and Utilization of Forest Products, Nanjing Forestry University, Nanjing 210037, China; 3 Jiangsu Province Key Laboratory of Green Biomass-Based Fuels and Chemicals, Nanjing Forestry University, Nanjing 210037, China

**Keywords:** cyanobacteria, carbon quantum dots, PVA, CNF, fluorescence, UV barrier, flexible

## Abstract

Blue luminescent carbon quantum dots (CQDs) were prepared from cyanobacteria by a hydrothermal method. The PL quantum yields of the obtained CQDs was 5.30%. Cyanobacteria-based carbon quantum dots/polyvinyl alcohol/nanocellulose composite films were prepared, which could emit bright blue under UV light. FTIR characterization showed that the composite films had hydroxyl groups on the surface and no new groups were formed after combining the three materials. The photoluminescence (PL) spectra revealed that the emission of the prepared CQDs was excitation dependent. Studies on the water resistance performance and light barrier properties of the composite films showed that they possessed higher water resistance properties and better UV/infrared light barrier properties. Therefore, we report the cyanobacteria-based carbon quantum dots/polyvinyl alcohol/nanocellulose composite films have the potential to be applied in flexible packaging materials, anti-fake materials, UV/infrared light barrier materials and so on.

## 1. Introduction

Cyanobacterial (CB) blooms found in many lakes, ponds and rivers have become a serious threat to human and animal health throughout the world [1]. Cyanobacteria are Gram-negative bacteria capable of producing a wide range of potent toxins—cyanotoxins—as secondary metabolites, which can cause acute and possibly chronic public health problems and fatal poisoning in aquatic and domestic animals [2,3]. Removing extensively accumulated cyanobacteria from the water is generally considered the most straightforward solution to CB blooms. As a result, scientists and entrepreneurs have paid close attention to the collected cyanobacteria. If the biomass in cyanobacteria can be reduced to a certain level, the problem of its environmental risk can be solved [4]. Given that cyanobacteria contain large amounts of plant proteins, amino acids and other extractable substances, people have tried to use the collected cyanobacteria as a biological resource to make value-added products, such as biofertilizer [5], high purity phycocyanin [1], activated carbon [6] and algae powder [7]. In this study, carbon quantum dots (CQDs) were extracted from collected cyanobacteria and used to produce fluorescent polyvinyl alcohol (PVA) films, which provides a new way to ameliorate cyanobacteria pollution.

Carbon quantum dots are a new carbon nanomaterial with a size less than 10 nm. They have become an emerging star in the field of carbon materials in a short time due to their high fluorescence [8], high solubility in water, unique photoluminescence, low toxicity, and excellent biocompatibility [9,10,11]. CQDs has shown great potential in several spheres including bio-imaging [12,13], chemical sensing [14], light-emitting diodes [15], photocatalysis and solar energy harvesting [16,17]. Up to now, the preparation methods for CQDs can be divided into two categories, the “top-down” and the “bottom-up” approaches. In recent years, one-step synthesis of CQDs has become the mainstream, among which hydrothermal methods have been some of the most widely used synthesis methods, and there are many materials can be used as its carbon source [14]. The CQDs synthesized from maltose by a hydrothermal method presented high electrocatalytic performance and electrical conductivity [9]. The CQDs extracted from lemon juice by a simple hydrothermal treatment could be applied to imaging plant cells [18]. Recently in 2019, Huang et al. stated that they had successfully prepared CQDs from wheat straw and bamboo via a one-pot hydrothermal treatment [19]. Zhang et al. also claimed to have successfully obtained CQDs from sodium citrate and carbamide, and produced high photostable epoxy polymerized CQDs luminescent thin films for display devices [20]. Meanwhile, Periyayya et al. developed flexible polymer films containing CQDs/N-doped ZnO nanoparticles with UV shielding capability of up to 47% [21]. In this study, we also used a hydrothermal method to extract CQDs from cyanobacteria.

Polyvinyl alcohol (PVA) is a highly polar water soluble polymer formed by the hydrolysis and polymerization of vinyl acetate, which was first prepared in 1924 by Herman and Haehnel [22,23]. PVA is a creamy or whitish, tasteless, odorless, nontoxic, biocompatible, thermostable, granular or powdered semi crystal line or linear synthetic polymer [24]. The amount of hydroxylation determines the physicochemical and mechanical attributes of the PVA [25]. PVA is a biodegradable polymer, and its degradability is enhanced through hydrolysis because of the presence of hydroxyl groups on the carbon atoms [26]. The complete dissolution of PVA in water is bound by its intrinsic properties, which require the water temperature to be at ~100 °C with a holding time of 30 min [27]. PVA is widely used in many areas like clinical applications, membrane fabrication and food packaging [28,29,30], because of its many attractive properties, such as biocompatibility, hydrophilicity, nontoxicity, biodegradability, as well as good mechanical properties, thermal stability, transparency, resistance to oxygen permeation and film-forming behavior [31,32,33]. This polymer is widely used by blending with other polymer compounds, such as biopolymers and other polymers with hydrophilic properties; it is utilized for various industrial applications to enhance the mechanical properties of films because of its compatible structure and hydrophilic properties [34]. Nonetheless, the heat resistance, water resistance and other barrier performance of PVA are not good enough, which limit its application. Many papers have focused on preparation of functional PVA-based composites by adding natural fibers to PVA. Zhong et al. used oil palm ash produced through the incineration process as a blending material with PVA to prepare thermoplastic film and found that the new film had better water resistance and degradation [35]. It is suggested that cellulose nanofibers (CNF) constitute ideal nanofillers to reinforce composites [36] due to their excellent properties such as low density, high tensile strength and biodegradability [37,38]. Tang et al. added CNF into oil well cement and then obtained CNF-OWC slurries with higher gel strength, yield stress and viscosity [39]. Wang et al. prepared CNF from peanut shells by chemical-mechanical treatments and an impregnation method and the obtained composite film exhibited high mechanical and thermal properties [40]. Qua et al. also fabricated PVA/CNF composite films with outstanding mechanical properties [41] using CNF extracted from flax fibers as the reinforcing phase. Chen et al. prepared CNF from wood powder (the softwood Hinoki cypress) by a simple grinding method and added CNF to polyacrylamide (PAM) gels which increased the compression stress of the obtained PAM/CNF by 6.8-fold as compared to that of pure PAM gel [42].

In conclusion, cyanobacteria can be used as carbon source to extract CQDs, which can be considered a new way to remedy cyanobacteria pollution. Carbon quantum dots fluorescent film is a new kind of photoluminescent functional optical film material. The fluorescence property is useful for future applications, such as an anti-fake packaging film, biological imaging and photocatalytic degradation [43]. PVA is widely used by blending with other biopolymers, such as CDQs and CNF is the basis of ideal nanofillers to reinforce composites. Therefore, we think it is an interesting topic to prepare fluorescent films by combining carbon quantum dots with PVA and CNF. However, research about PVA/CNF/CQDs composite films has not been publically reported. In this study, we studied the effect of CQDs load on the properties of the prepared PVA/CNF/CQDs composite films in detail.

## 2. Materials and Methods

### 2.1. Materials

Propylene glycol, polyvinyl alcohol, benzene, ethanol, sodium chlorite, glacial acetic acid, sodium hydroxide and hydrochloric acid used in this experiment were purchased from Nanjing Chemical Reagent Co., Ltd. (Nanjing, China). Cyanobacteria (CY) salvaged from Taihu Lake (Wuxi, China) was firstly air-dried for 48 h and then oven-dried at 60 °C in an oven for 48 h (the moisture content, <20%). CY powder with 70 mesh was obtained by screening. The agricultural and forestry waste for extracting CNF, coconut palm petiole, was provided by Hainan Kunlun New Material Science & Technology Co., Ltd. (Haikou, China). After being cleaned with water and air-dried, the petiole was broken into small particles with a size of 2–3 × 6–7 (mm) by a L-905 shredder, ground into woody powder with a FZ102 miniature plants grinder (TAISITE instrument Co., Tianjin, China), and sieved using a sieve with 60 meshes (Zhang Xing Sand Screen Factory, Shangyu, Zhejiang Province, China).

### 2.2. Preparation of CNF

Cellulose nanofibers was extracted from coconut palm petiole and the steps were referred to the report of Zhao et al. [44].

### 2.3. Synthesis of CQDs

Cyanobacterial carbon quantum dots was synthesized with a hydrothermal method which was referred to Wang et al. [45]. The CY powder (1.5 g) was treated in a mixture of purified water/glycerol (3:1 by volume) under stirring at room temperature for 20 min. Then the mixture was transferred into a polytetrafluoroethylene-equipped stainless-steel autoclave (100 mL) and heated at 200 °C for 8 h in an oil bath. The resulted dark brown product which consisted of CQDs solution and black residue was filtered with a piece of microporous membrane (0.22 µm, pore size), resulting in cyanobacterial carbon quantum dots.

The solution drying method was used to calculate the concentration of the prepared cyanobacterial CQDs according to Equation (1):

(1) $$C=\frac{M_{2}-M_{1}}{V}$$

where *C* refers to the concentration of CQDs, *M*_1_ refers to total mass (mg) of clean slides and cover slides and *M*_2_ refers to the mass (mg) of dried carbon quantum dots dispersion with slide and cover glass, and V represents volume of CQDs. Finally, the concentration of CQDs was 175 mg/mL.

### 2.4. Preparation of Films

CQDs solution (175 mg/mL, 0.1 mL), PVA solution (10%, 100 mL) and CNF solution (1%, 100 mL) were mixed in a breaker under stirring for 30 min, and then ultrasonically processed (XO-1200, Nanjing Xianou Instrument Manufacturing Co., Ltd., Nanjing, China) at 60% power for 20 min. After that, the mixture was transferred into a Petri dish and then oven-dried (101-2BS, Beijing Hengnuolixing Technology Co., Ltd., Beijing, China) at 40 °C for 24 h, resulting in a polymer film labeled as No. 2. Likewise, the composite films named as No. 3, No. 4, No. 5, No. 6 and No. 7 were prepared in the same way by mixing 0.3, 0.5, 1.2, 2.0 and 4.0 mL CQDs, respectively. No. 1 without CQDs was the control for No. 2–No. 7.

### 2.5. Characterization

A ZF-1 three-use ultraviolet analyzer (Hangzhou Qiwei Instrument Co., Ltd., Hangzhou, China) was used in this study to see if the produced CQDs and the corresponding films had fluorescence effects. Photoluminescence performance of the CQDs and films was investigated with a fluorescence spectrometer (Model LS-55, PerkinElmer, Waltham, MA, USA). The ultraviolet-visible spectroscopy (UV-vis) absorption spectra were recorded on a Lambda 950 spectropolarimeter (PerkinElmer). FT-IR spectra of the film samples were acquired with a Fourier transform spectrometer (NICOLET IS10, Thermo Scientific, Inc., Waltham, MA, USA) operating in the Smart iTR diamond ATR mode and the range from 500 cm^−1^ to 4000 cm^−1^. Barrier properties of the films to light (200–2500 nm) was tested by a U-4001 spectrophotometer (Hewlett-Packard Co., Santa Clara, CA, USA). A cold field emission scanning electron microscope (Regulus 8200, Hitachi, Tokyo, Japan) was used to analysis the fracture morphology of the films. The water resistance of films was expressed by the water absorption. The samples with a size of 2 cm × 2 cm were first dried at 100 °C for 24 h, then weighed (W_1_) and soaked in distilled water (50 mL) for 24 h. After drying its surface, the sample was weighted again (W_2_), and the water absorption (A) of the sample was calculated by Equation (2):

(2) $$A=\frac{W_{2}-W_{1}}{W_{1}}\times 100\%$$

## 3. Results and Discussion

### 3.1. Characterization of CQDs

**Figure 1:**
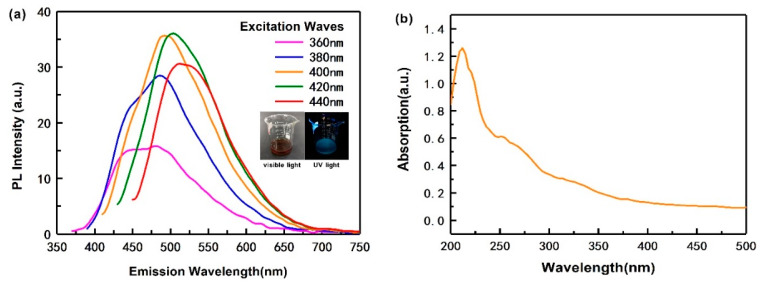
(**a**) Emission spectrum of the as-obtained CQDs at different excitation wavelengths (360–440 nm); (**b**) UV-vis absorption spectra of CQDs.

The optical properties of the as-obtained CQDs under visible and UV light are shown in Figure 1a (inset). The CQDs appears brown and bright blue under visible and UV light, respectively. Even more interesting, in Figure 1a, when the wavelength of excitation increases from 360 to 440 nm, its emission intensity is different and the wavelength of the strongest emission peak of the CQDs is 450−525 nm. Moreover, when the excitation wavelength was 420 nm, the CQDs presented the strongest fluorescence intensity (36 a.u.), and a corresponding emission wavelength is 512 nm. This indicates that the PL spectrum of the CQDs displays excitation wavelength-dependent features. It means that the emission wavelength can be adjusted by changing the excitation wavelength. This property is useful for the future applications, such as an anti-fake packaging film, biological imaging and photocatalytic degradation [43]. This is similar to some of the other CQDs extracted from ascorbic acid [14] and soot [46]. The reasons why this phenomenon exists are still controversial. Lau et al. [47] and Xiao et al. [48] believed that the presence of different functional groups on the surface of carbon quantum dots can lead to multiple emission centers, with different ones dominating at different excitation wavelengths. Si et al. [49] further argued this point that if the functional groups on the surface of the carbon quantum dots are diverse, the emission centers will appear to be multiple, and the main emission center will change with the change of the excitation wavelength, which appears as excitation dependence. Zhang et al. [50] used (*N*-(2-aminoethyl)-3-aminopropyl) tris-(2-ethoxy) silane (KH791) as catalyzer, stabilizing and passivation agent, the prepared CQDs can be self-assembled in solution and exhibit excitation wavelength independent PL property. In addition, silane KH791 helps to enhance PL intensity of CQDs.

The PL quantum yields (QY) of CQDs was calculated by Equation (3) [51]:

(3) $$Q_{sam}=Q_{ref}\;\frac{I_{sam}A_{ref}N_{sam}^{2}}{I_{ref}A_{sam}N_{ref}^{2}}$$

where, *Q_sam_* and *Q_ref_* is the QY of the sample and the reference; *A_sam_* and *A_ref_* is the absorbance of the sample and the reference at an excitation wavelength; *N_sam_* and *N_ref_* is the refractive indices of the sample and the reference medium; *I_sam_* and *I_ref_* is the integrated fluorescence intensities of the sample and the reference. Here, quinine sulfate was used as the reference (QY = 54%) [52]. According to the PL spectra of the CQDs and the quinine sulfate, the QY of our CQDs is 5.30%.

The UV-vis absorption spectrum of the CQDs is shown in Figure 1b. As shown, the CQDs have two obvious absorption peaks, located at 212 and 251 nm. The strong absorption peak at 212 nm is assigned to the presence of cysteine or the effect of the carbon skeleton compared to the UV-vis absorption of pure cysteine and carbon skeleton, respectively [9]. The absorption peak at 251 nm is caused by π–π* transitions of the conjugated system [9].

### 3.2. FTIR Characterization of the PVA/CNF/CQDs Films

**Figure 2:**
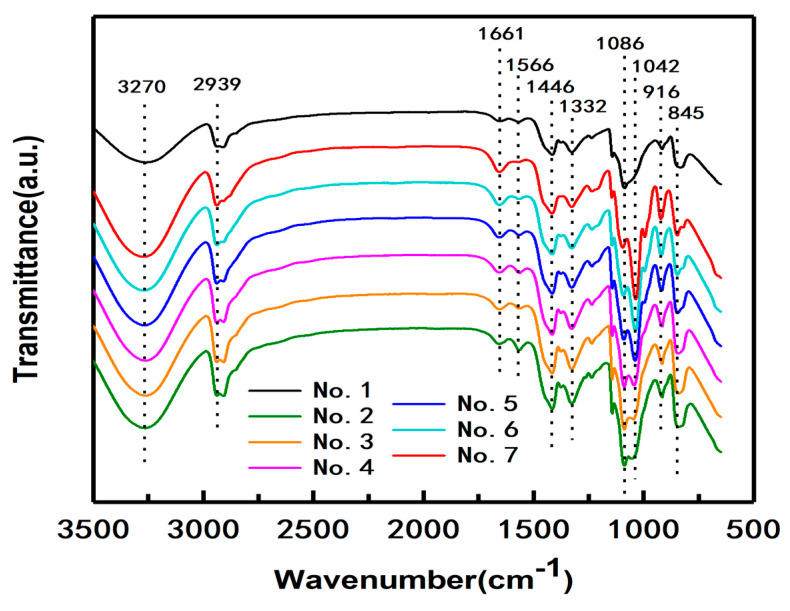
FTIR spectra of the composite films with different CQDs content.

Figure 2 shows the FTIR spectra of the produced PVA/CNF/CQDs films with different CQDs content as listed in Table 1. In the spectrum of the composite film No. 1 without CQDs, stretch vibrations of O–H, C–H, and C=O, are at 3270 cm^−1^, 2939 cm^−1^ [53], and 1661 cm^−1^ [54], respectively. Specifically, the absorption peak at 1566 cm^−^^1^ is associated with the C=C stretching vibration [55]. After the addition of CQDs, the intensity of the peak at 1566 cm^−1^ slightly increases, and then the intensity of the absorption peak gradually decreases with the increase of CQDs content. One reason may be related to the increasing amount of C=C, which exist in the skeleton of the CQDs [45]. The other reason for the result is possible that thermal treatment tends to facilitate the precursor to undergo dehydration, decomposition, polymerization and carbonization, and thus leads to the formation of carbon skeletons [45]. The absorption peak at 1446 cm^−^^1^ is ascribed to O–H and C–H bending vibrations [45], and the peak at 1332 cm^−^^1^ is attributed to O–H and C–H shaking vibrations. The absorption peaks at 1086 and 1042 cm^−^^1^ are attributed to the stretching vibrations of C–O–C and C-O, respectively. With the increase of CQDs content, the intensity of the absorption peak at 1042 cm^−^^1^, which is the characteristic of cyanobacteria-derived CQDs, gradually increases [45,56]. The absorption peaks at 916 and 845 cm^−^^1^ correspond to –CH_2_ and C–C bending vibrations, respectively. Therefore, the FTIR spectrum of sample No. 1 indicates that it mainly contains functional groups such as O–H, C–H and C=O.

The spectra of samples No. 2–No. 7 with CQDs are similar to that of No. 1, indicating that the introduction of CQDs into the composite PVA/CNF does not add new functional groups, that is to say, there are no new chemical reactions between CQDs and PVA/CNF.

### 3.3. PL Spectra of the PVA/CNF/CQDs Films

**Figure 3:**
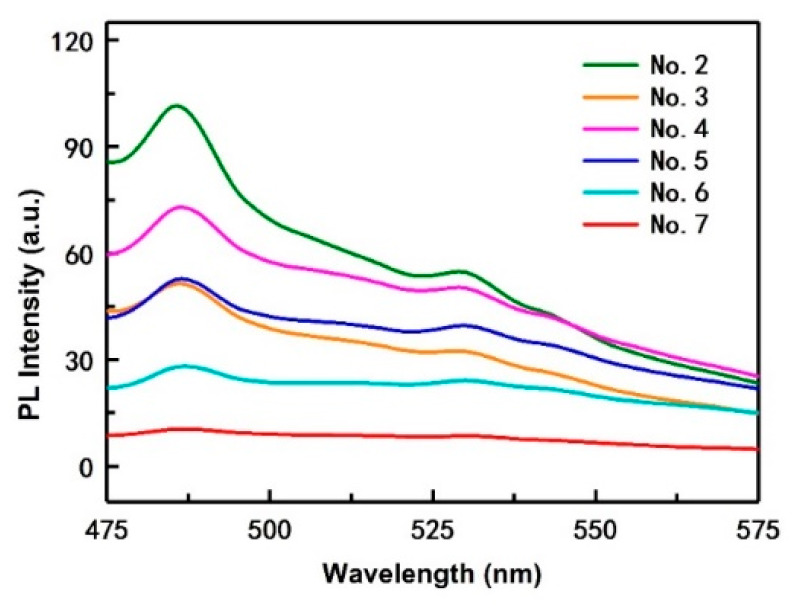
PL spectra of the composite films with different CQDs content at 420 nm excitation wavelengths.

Figure 3 shows the PL spectra of the composite films with different CQDs content. As shown in Figure 1a, when the excitation wavelength is 420 nm, the emission intensity of the prepared CQDs is the highest. Therefore, here we selected the light with a wavelength of 420 nm to excite the films to study their photoluminescent properties.

It is observed that the fluorescence spectra of all PVA/CNF/CQDs films present a similar changing trend. In general, the intensity of the strongest emission of film No. 2 is higher than that of a CQD solution. According to [57], the fluorescence of PVA at excitation wavelengths less than 420 nm is very weak. The excitation wavelength selected in this study is exactly 420 nm, and the amount of PVA is certain, so PVA has almost no effect on the fluorescence of the composite film. According to another paper [58], introducing CNF into the PVA/CQDs films can increase emission intensity, which explains why the intensity of the strongest emission of film No. 2 is higher than that of CQD solution.

With the increase of CQDs content, the intensity of the strongest emission of the PVA/CNF/CQDs films gradually weakens. This shows that the CQD content has a significant effect on the attenuation of the fluorescence intensity of composites, consistent with the conclusions of Ling [59]. One possible reason may be due to the π–π * and n–π * electron transitions improve after adding CQDs in PVA/CNF films, thus weakening the fluorescence intensity of the films [60]. The other possibility is due to the increase of electron-withdrawing group (carbonyl group) in the films, which can weaken the fluorescence [61]. For all PVA/CNF/CQDs films, when the excitation wavelength is 420 nm, the wavelength corresponding to the strongest emission is less than 512 nm, which is the wavelength for the CQDs itself, as shown in Figure 1a. We think that PVA and CNF reduce the density of the CQDs’ luminous centers which causes the observed blue shift in the spectrum.

Moreover, the fluorescence spectra are clearly visible with convex peaks in the emission wavelength of 475 to 500 nm, which is the lipid oxidation fluorescence peak. The reason is that 2p2 non-bonding electrons of the oxygen atom occurs n–π * electron transition in the free hydroxyl group of the molecular conformation of PVA [61].

### 3.4. Water Resistance Performance of the PVA/CNF/CQDs Films

**Figure 4:**
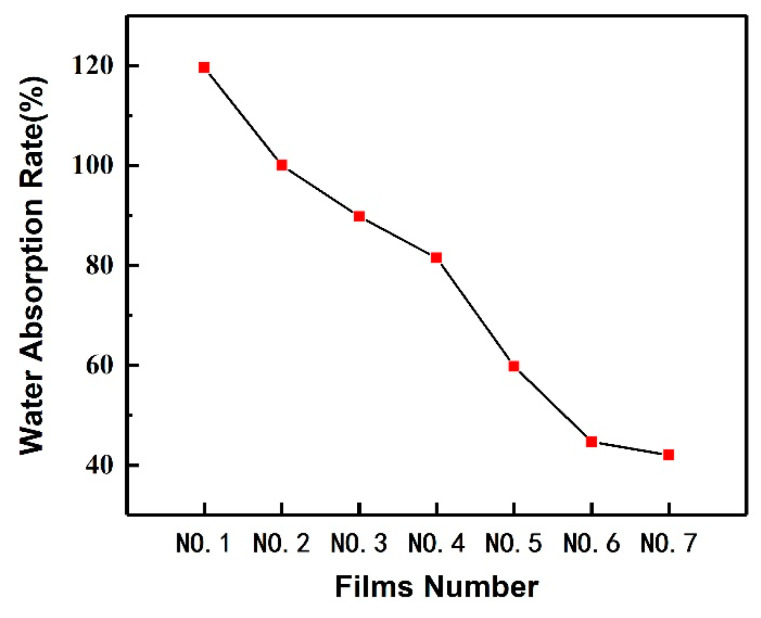
Water absorption performance of the films.

The water absorption performance of the prepared PVA/CNF and PVA/CNF/CQDs composite films is shown in Figure 4. In the PVA/CNF matrix, although hydrogen bonds are easily formed between CNF and PVA, there are still many free hydroxyl groups, which is confirmed in Figure 2, resulting in a high water absorption rate (119.6%). With the increase of CQDs from 0.1 to 4 mL, the water absorption rate of the PVA-based film decreases significantly from 119.6% to 42.0%, indicating that introducing a certain amount of CQDs into PVA/CNF matrix can improve the water resistance performance of the PVA-based films. One reason may be due to the reduction of porosity [62]. The other reason is when CQDs with many carboxyl groups are introduced, the decrease of free hydroxyl radicals is due to the reaction between hydroxyl and carboxyl groups. This leads to the decrease of water absorption of the films [63].

### 3.5. Light Barrier Property of the PVA-Based Films

**Figure 5:**
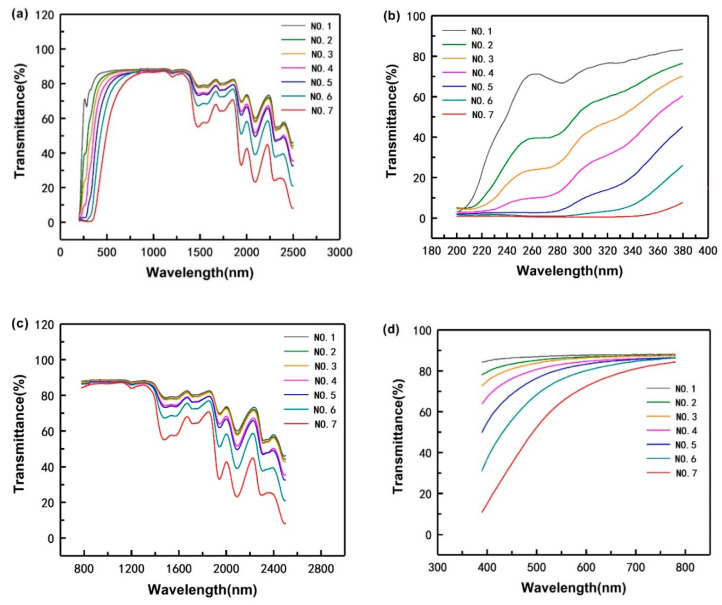
PVA-based composite’s light transmittance to the light at wavelength between 200 and 2500 nm (**a**); to UV light (**b**); to infrared light (**c**); to visible light (**d**).

The light transmittance of the PVA-based composites to the light at wavelengths between 200 and 2500 nm is shown in Figure 5a. The transmittance of the samples with CQDs (No. 2–No. 7) is lower than that of the sample without CQDs (No. 1), suggesting that the light barrier properties of the PVA/CNF films can be improved by introducing CQDs into PVA/CNF matrix.

Furthermore, in order to investigate the barrier performance of the composite to light with different wavelengths, we described Figure 5a in sections according to different wavelengths, as shown in Figure 5b–d. In the range of ultraviolet light (wavelength ≤380 nm) and infrared light (wavelength ≥780 nm), compared with sample No. 1 without CQDs, the transmittance of composite films with CQDs (No. 2–No. 7) is significantly reduced; however, in the range of visible light, introducing CQDs into PVA/CNF does not significantly enhance the barrier performance of the composite. It indicates that in PVA/CNF/CDQs films, the improvement of light resistance caused by CQDs is mainly concentrated in the ultraviolet regions. As explained in Section 3.1, CQDs have two obvious absorption peaks in the ultraviolet region, which means CQDs can absorb ultraviolet light. Therefore, with the increase of CQDs content in matrix, the UV light barrier properties of the composite are getting better and better, indicating that the greater the amount of CQDs in the PVA/CNF matrix, the more obvious the improvement of the composites’ UV light barrier.

## 4. Conclusions

Cyanobacterial blooms represent a serious threat to the health of humans and animals around the world. In this work blue luminescent carbon quantum dots were prepared from cyanobacteria by a hydrothermal method. The PL quantum yields of the obtained CQDs was 5.30%. Cyanobacteria-based carbon quantum dots/polyvinyl alcohol/nanocellulose composite films were prepared, which could emit bright blue under UV light. The introduction of CQDs into the PVA/CNF film is expected to improve the water resistance performance and achieve good UV-light-barrier, anti-fake and high trasparency film materials, which have promising applications in the flexible packaging field.

FTIR characterization showed that the composite films had many hydroxyl groups on the surface and the spectra of samples No. 2–No. 7 with CQDs are similar to that of No. 1, indicating that the introduction of CQDs into the composite PVA/CNF does not add new functional groups, that is to say, there are no new chemical reactions between CQDs and PVA/CNF.

The PL spectra revealed that the emission of prepared CQDs was excitation dependent and with the increase of CQDs content, the intensity of the strongest emission of the PVA/CNF/CQDs films gradually weakens.

Studies on the water resistance performance showed that the composite films possessed higher water resistance properties. With the increase of CQDs from 0.1 to 4 mL, the water absorption rate of the PVA-based film decreases significantly from 119.6% to 42.0%, indicating that introducing a certain amount of CQDs into the PVA/CNF matrix can improve the water resistance performance of the PVA-based films.

In the study of the light barrier properties of the PVA-based films, we found that with the increase of CQDs content in the matrix, the light barrier effect of the composite is getting better and better, indicating that the greater the amount of CQDs in PVA/CNF matrix, the more obvious the improvement of the composites’ light barrier properties.

Therefore, we report the cyanobacteria-based carbon quantum dots/polyvinyl alcohol/nano-cellulose composite films have the potential to be applied in flexible packaging materials, anti-fake materials, UV/infrared light barrier materials and so on.

## Figures and Tables

**Table 1:** The experimental scheme and formula of the films.

Film No.	CQDs/mL *	PVA/mL *	CNF/mL *
1	0	100	100
2	0.1	100	100
3	0.3	100	100
4	0.5	100	100
5	1.2	100	100
6	2.0	100	100
7	4.0	100	100

* The volume of sample solution.
